# Evaluation of Toxic Elements in Commercial Dried Mushrooms from the Polish Market: Exposure Assessment and Health Risk Characterization

**DOI:** 10.3390/molecules31111865

**Published:** 2026-05-29

**Authors:** Joanna Domagalska, Monika Rusin, Danuta Rogala, Klaudia Gut-Pietrasz, Marta Buczkowska

**Affiliations:** 1 Department of Environmental Health, Faculty of Public Health in Bytom, Medical University of Silesia in Katowice, 18 Piekarska Street, 41-902 Bytom, Poland; mrusin@sum.edu.pl (M.R.); drogala@sum.edu.pl (D.R.); kgut@sum.edu.pl (K.G.-P.); 2 Department of Occupational Medicine and Health in Department of Chronic Diseases and Civilization-Related Hazards, Faculty of Public Health in Bytom, Medical University of Silesia in Katowice, 18 Piekarska Street, 41-902 Bytom, Poland; mbuczkowska@sum.edu.pl

**Keywords:** toxic elements, food safety, environmental monitoring, edible mushrooms, dietary exposure, toxicological assessment

## Abstract

Edible mushrooms are highly valued for their nutritional properties, yet their exceptional capacity to bioaccumulate heavy metals necessitates rigorous safety assessments of commercially available products. This study evaluates the concentrations of Cd, Pb, Hg, and As in 164 samples of commercial dried mushrooms to assess consumer health risks. Analysis of six species revealed that contamination levels are significantly influenced by taxonomic factors, supplier variability, and certification status. While median concentrations across most species remain within safety thresholds, *Boletus edulis* and *Suillus* spp. exhibited the highest accumulation of Cd and As, respectively. A critical finding was an incidental, extreme As concentration (369.048 mg·kg^−1^) in *Imleria badia*, resulting in a Hazard Index (HI) of 63.57, far exceeding the safety limit of 1. Statistically significant differences (*p* < 0.01) were observed between organic and conventional products, with certified samples showing lower Hg and As levels. Although moderate consumption of most batches is safe, the high variability between producers and the presence of toxicological outliers underscore the urgent need for rigorous monitoring of harvesting areas. These findings suggest that organic certification enhances food safety and highlight the necessity for targeted nutritional guidelines.

## 1. Introduction

In recent years, there has been growing consumer interest in natural products, which are perceived as safe, nutritious, and beneficial to health [1,2]. This trend encompasses food sourced from the natural environment, including wild-growing edible mushrooms, which constitute a significant component of the traditional diet in numerous European countries, particularly in Central and Eastern Europe [3,4,5]. The consumption of mushrooms constitutes an important dietary source of flavour-enhancing components and bioactive substances (particularly polysaccharides, phenolic antioxidants and essential trace elements) [3,4,5,6,7].

It has been demonstrated that certain organisms, when present in their natural environment, possess the capacity to accumulate pollutants in soil, water and air. It is evident that mushrooms’ fruiting bodies merit particular consideration due to their distinctive morphological structure and physiological characteristics [8,9]. These characteristics are conducive to the uptake and accumulation of trace elements, including heavy metals [8,9,10,11]. The development of an extensive mycelium network, which penetrates significant volumes of substrate, has been shown to increase the surface area of contact with the soil. This, in turn, enables the effective uptake of both nutrients and pollutants. The cell walls of these organisms contain various compounds, including chitin, glucans, and melanin, which have the capacity to bind metal ions [8,9,10]. Consequently, fruiting bodies can serve as a pivotal conduit in the transfer of heavy metals from the environment to the human food chain. However, the capacity to absorb and accumulate heavy metals varies, and this capacity is dependent on the species of mushroom, owing to the different physiological characteristics of individual species [9,10,11]. Because these toxic elements are chemically integrated into the fungal biomass and sequestered within the structural components of the fruiting body, they are not significantly removed by standard domestic processing such as washing or soaking. While thermal processing may induce minor leaching of certain water-soluble fractions, the majority of the elemental load remains within the tissue. Consequently, toxicological risk assessments for mushrooms are typically based on the total concentration in the dry matter to provide a robust and conservative safety margin for public health [8,9,10,11]. Furthermore, the capacity to absorb and accumulate heavy metals varies, and this capacity is dependent on the species of mushroom, owing to the different physiological characteristics of individual species [9,10,11].

In the context of food safety, the traceability of a product’s origin, in particular the area where it was sourced, is of fundamental importance. The concentration of heavy metals in mushrooms is contingent on the degree of environmental pollution, particularly in soils adjacent to industrial areas, transport routes, or areas with a history of industrial emissions [8,12,13]. In the context of products designated as ecological, specific control procedures are in place to ensure the method of production and the quality of the raw material meet defined standards. However, for other products available in retail outlets, consumers usually only have information about the address of the producer or packager, which is not the same as the place where the mushrooms were harvested [8,12,13]. In practice, this means that there is a lack of complete knowledge about the environmental conditions of their origin and, therefore, their potential exposure to pollution.

Among environmental pollutants, heavy metals such as arsenic (As), cadmium (Cd), lead (Pb), and mercury (Hg) are of particular importance in the context of food safety [14,15,16,17,18]. These elements can occur in the environment as a result of both natural processes and anthropogenic activities, including industrial emissions, fossil fuel combustion, metallurgy, and transport [14,15]. These substances are distinguished by their environmental persistence, bioaccumulation capacity, and the potential to enter the food chain, thereby constituting a significant health risk factor [14,15,16,17,18].

It is evident that As, in particular its inorganic compound form, has been designated by the International Agency for Research on Cancer (IARC) as a human carcinogen (Group 1) [19]. There is sufficient evidence to suggest a correlation between chronic exposure and the development of various types of cancer, including skin cancer (mainly non-melanoma), lung cancer, and bladder cancer [19,20,21,22]. Extensive documentation exists which demonstrates that chronic exposure to arsenic can result in a variety of cutaneous alterations, including hyperkeratosis and pigmentation disorders. These conditions are widely regarded as precancerous pathologies [19,20,21]. As has been demonstrated, the substance exerts genotoxic effects, influences oxidative stress processes, and contributes to DNA repair disorders [19,20,21,22,23].

Cd has also been classified as a human carcinogen (Group 1) [19]. The extant literature indicates a primary correlation between occupational and environmental exposure and an elevated risk of lung cancer [19,24,25]. In addition, epidemiological data has been interpreted to suggest a possible correlation between exposure and prostate and kidney cancer [19,24,25,26]. The predominant site of Cd accumulation is the kidneys, with a biological half-life ranging from several to several dozen years, resulting in renal tubule damage and chronic nephropathy [25,26,27,28]. The mechanisms by which the substance exerts its effects include the induction of oxidative stress, the dysregulation of apoptosis, and interactions with metallothioneins [24,25,26,27,28].

Pb and its inorganic compounds are classified by the IARC as probably carcinogenic to humans (Group 2A). The epidemiological evidence suggests a potential association with brain, kidney, and stomach cancers [25,27,29,30,31,32]. However, the strength of these associations is comparatively weaker than that observed for As or Cd. It has been demonstrated that Pb exerts a predominant neurotoxic effect, particularly during periods of development, thereby inducing the onset of permanent cognitive and behavioural disorders [29,30,31]. Moreover, the potential consequences of this phenomenon extend to the haematopoietic and cardiovascular systems, with the capacity to induce calcium (Ca) metabolism disturbances [29,30,31,32].

The toxicity of Hg and its compounds is dependent on their chemical form. Methylmercury (MeHg) is regarded as a highly potent neurotoxin capable of crossing the blood–brain and –placental barriers, thereby posing a particular risk to foetuses and children [27,33,34,35]. According to the IARC classification system, MeHg is categorised as Group 2B, indicating a possible carcinogenic effect in humans [27,33,34,35,36]. However, the available data on the carcinogenicity of other Hg compounds is limited. Nonetheless, the prevailing consequences of chronic exposure manifest chiefly as neurological and immunological disorders, and renal deterioration [27,33,34,35,36].

It is imperative to monitor the content of these elements in food, as there is documented evidence of their toxic effects, and in the case of some elements, their carcinogenic potential has been clearly confirmed. This monitoring constitutes a pivotal component in evaluating the risk to population health [8,12,13].

Edible wild-growing mushrooms constitute a highly seasonal and perishable raw material due to their high water content, which can reach 85–90% of the mushroom’s fresh weight [37,38,39]. Consequently, they are predominantly traded and consumed in dried form in commercial markets, a practice that facilitates long-term storage and distribution, irrespective of the harvest season. The drying process leads to a concentration of the components present in the dry mass, including trace elements and potential contaminants [37,38,39].

In the scientific literature, it is common practice to determine the heavy metal content in material dried under laboratory conditions [40,41,42,43]. The results most often refer to the dry weight of mushroom samples. However, the majority of extant research in this field focuses on samples collected from precisely defined field locations, which allows for a direct correlation to be established between concentrations and environmental pollution in a given area [40,41,42,43]. In less frequent cases, dried mushroom products offered for retail sale are analysed, in which case the actual location of sample collection is not clearly identifiable. In practice, consumers purchase products bearing the name of the producer or packager, yet this fails to provide information regarding the environmental context in which the mushrooms were harvested. The absence of this knowledge hinders the capacity to evaluate the potential exposure resulting from the provenance of the raw material, particularly in the context of the potential harvesting of mushrooms from areas characterised by elevated levels of anthropogenic pollution, including regions adjacent to industrial sites or extensive transport infrastructure.

In view of the aforementioned conditions, it is reasonable to conduct an analysis of the heavy metal content in dried edible mushrooms available in retail stores, reflecting the actual exposure of consumers. The extant literature focuses primarily on samples collected from specific environmental locations, while data concerning the heavy metal contamination of commercial products available on the domestic market remains limited.

Therefore, the primary objective of this study was to evaluate the concentrations of toxic elements (Cd, Pb, Hg, and As) in various species of commercially available dried mushrooms sourced from the Polish market and to assess the associated health risks for consumers. Furthermore, this research aimed to determine the influence of the raw material supplier and organic certification status on the safety profile of the final products. By applying standardized risk assessment indices (ADD, HQ, and HI), this study seeks to identify potential toxicological hazards and provide practical insights for food safety management and consumer protection within the Polish forest products sector.

This study expands the current state of knowledge by evaluating multiple differentiating factors of the commercial market, such as species-specific accumulation patterns and the influence of organic vs. conventional production systems. By focusing on processed products (dried mushrooms) from the retail chain, we provide a representative health risk assessment that accounts for the variability in commercial sourcing and producer-level quality standards.

## 2. Results

A total of 164 samples, representing six species of edible mushrooms sourced from eight Polish producers, were subjected to analysis. A detailed characterization of the research material is presented in Table 1.

The concentrations of heavy metals in the analyzed mushroom samples exhibited distinct variability across the studied elements, species, and producers (Table 2, Table 3, Table 4 and Table 5).

Hg concentration averaged 0.055 mg·kg^−1^ (median; range: 0.020–0.134), with values below the limit of quantification (LOQ) recorded in 16 samples (9.76%). The lowest concentration was below <0.002 mg·kg^−1^ (substituted with 0.001 mg·kg^−1^), while the maximum reached 4.240 mg·kg^−1^. For Cd, the median concentration was determined at 0.595 mg·kg^−1^ (0.170–1.345). In three samples (1.83%), levels were below the LOQ of <0.01 mg·kg^−1^ (substituted with 0.005 mg·kg^−1^), with a maximum concentration of 9.860 mg·kg^−1^. Pb concentrations were generally lower, with a median value of 0.050 mg·kg^−1^ (0.050–0.271). A significant proportion of the material, totaling 96 samples (58.54%), was characterized by levels below the LOQ of <0.1 mg·kg^−1^ (substituted with 0.05 mg·kg^−1^). The highest Pb concentration reached 2.237 mg·kg^−1^. As was present at a median level of 0.983 mg·kg^−1^ (0.33–2.492). In 75 samples (45.73%), concentrations did not exceed the LOQ of <0.66 mg·kg^−1^ (substituted with 0.33 mg·kg^−1^). However, the maximum recorded content was substantially higher, reaching 369.048 mg·kg^−1^. The maximum arsenic concentration (369.048 mg/kg) observed in one sample of *Imleria badia* was confirmed through triple re-analysis to rule out analytical error.

Analysis of the cultivation system revealed differences in the levels of specific metals. Significantly higher concentrations of Hg and As were observed in samples from conventional cultivation. For Cd and Pb, median concentrations were slightly higher in the organic material; however, these differences did not reach statistical significance (Table 6).

Inter-species differentiation was evident for all analyzed elements. The highest concentrations were most frequently observed in *Boletus edulis*, *Leccinum aurantiacum*, and *Suillus luteus*, whereas *Cantharellus cibarius* and *Agaricus bisporus* were characterized by lower concentrations, as confirmed by the medians presented in Table 2, Table 3, Table 4 and Table 5. Among producers, subgroups with significantly different concentrations were identified through post hoc tests; the letter coding in Table 2, Table 3, Table 4 and Table 5 provides a synthetic illustration of these relationships.

Spearman’s rank correlation analysis revealed statistically significant relationships between selected pairs of elements (*p* < 0.05). Moderate positive correlations were observed between cadmium and lead (R = 0.533), mercury and cadmium (R = 0.508), and mercury and lead (R = 0.466). A weak positive correlation was found between mercury and arsenic concentrations (R = 0.215), while a weak negative relationship was noted for the correlation between cadmium and arsenic (R = −0.262) (Table 6).

### Health Risk Assessment

The analysis of average daily dose (ADD) and hazard quotient (HQ) revealed significant differences among the studied species of wild-growing edible mushrooms (Table S1—Supplementary Materials). Assuming a consumption level of 3.6 g/day, the highest mean hazard index (HI) values—representing the sum of HQ coefficients for individual elements—were recorded for: *Suillus luteus* (HI = 0.9359), *Leccinum aurantiacum* (HI = 0.2752), and *Cantharellus cibarius* (HI = 0.2212). The lowest health burden was observed for the cultivated control species *Agaricus bisporus*, for which the HI reached only 0.0595.

Notably, in the case of maximum values for *Imleria badia*, the HI soared to 63.5673, driven by an extremely high arsenic (As) concentration of 369.048 mg·kg^−1^. This outlier value suggests a localized point-source of environmental contamination or an exceptional bioaccumulation capacity of the individual specimen, posing a potential acute toxicological risk. Regarding the status of the samples, mushrooms harvested from non-certified wild areas were characterized by a higher mean HI (0.2507) compared to those from certified organic areas (0.1111).

Analysis of the results in relation to the producers (Table S2—Supplementary Materials) revealed that the level of health risk is closely correlated with the specific source of the raw material. The highest mean Hazard Index (HI) values were recorded for Producer 5 (HI = 0.5892) and Producer 6 (HI = 0.4816), whereas the lowest cumulative risk was observed for Producer 4 (HI = 0.1119). Regarding maximum values, products from Producer 7 emerged as a critical point, where the HI reached a level of 63.7559. Similar to the species-specific analysis, this was primarily attributed to an incidental, extremely high concentration of arsenic (369.048 mg·kg^−1^), as well as elevated cadmium levels found in samples from other suppliers (e.g., Producer 3, where Cd reached 9.860 mg·kg^−1^).

**Figure 1:**
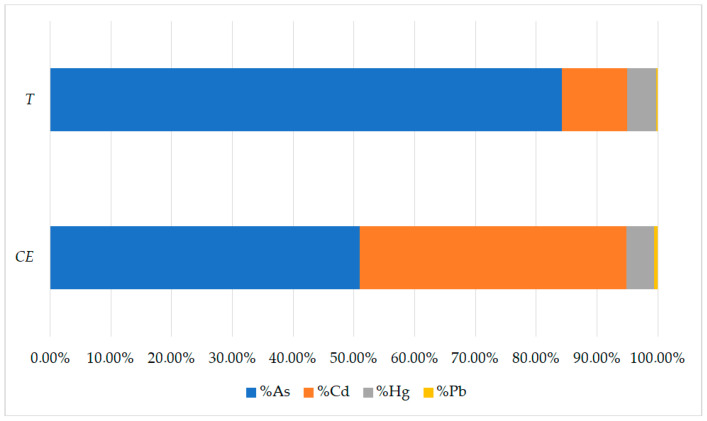
Percentage contribution of Hg-, Cd-, Pb-, and As-specific HQ values to the total HI, stratified by cultivation type. Note. CE—certified organic cultivation; T—traditional wild-growing mushrooms.

**Figure 2:**
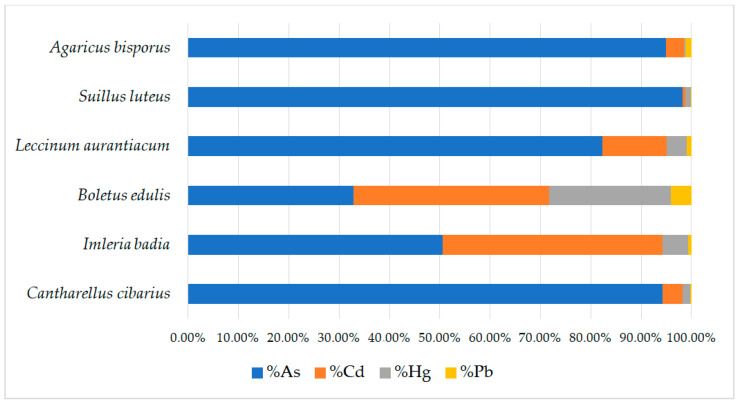
Percentage contribution of Hg-, Cd-, Pb-, and As-specific HQ values to the total HI, stratified by producer.

**Figure 3:**
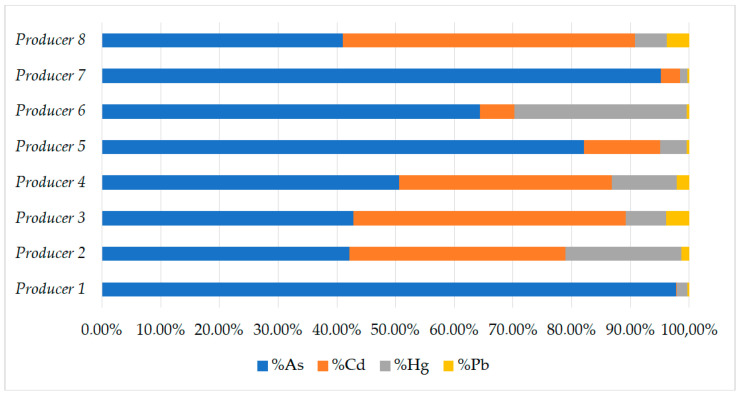
Percentage contribution of Hg-, Cd-, Pb-, and As-specific HQ values to the total HI, stratified by mushroom species.

The relative contribution of Cd-, Pb-, Hg-, and As-specific HQ values to the total Hazard Index is presented in Figure 1, Figure 2 and Figure 3, according to cultivation type, manufacturer, and mushroom species, respectively.

These findings indicate that while the mean consumption of most studied mushrooms does not exceed the safety threshold (HI < 1), the significant variability between producers and individual batches (maximum values) may pose a substantial health risk to consumers. This risk is particularly pronounced in terms of exposure to arsenic and cadmium.

## 3. Discussion

Edible mushrooms, particularly ectomycorrhizal species, serve as natural bioindicators of environmental pollution due to their exceptional capacity for heavy metal bioaccumulation within their fruiting bodies. The present study, encompassing 164 samples of dried wild-growing mushrooms, revealed median concentrations of 0.595 mg·kg^−1^ d.w. for Cd, 0.050 mg·kg^−1^ d.w. for Pb, 0.055 mg·kg^−1^ d.w. for Hg, and 0.983 mg·kg^−1^ d.w. for As. These results demonstrate a strong dependence of contamination levels on the species, further supporting the hypothesis that taxonomic factors and ecological strategies play a pivotal role in shaping the chemical profile of the raw material [41].

Regarding cadmium, the highest accumulation was observed in *Boletus edulis* (mean: 1.30 mg·kg^−1^ d.w.; maximum: 9.860 mg·kg^−1^ d.w.) and *Imleria badia* (syn. *Xerocomus badius*; 0.95 mg·kg^−1^ d.w.). The obtained mean values are lower than those reported by Orywal et al. [44] for commercial dried mushrooms in Poland (1.98 mg·kg^−1^ d.w. for *B. edulis* and 1.15 mg·kg^−1^ d.w. for *X. badius*). Conversely, Gałgowska and Pietrzak-Fiećko [42] recorded Cd levels of 2.151 ± 0.066 mg·kg^−1^ d.w. and 0.615 ± 0.020 mg·kg^−1^ d.w. in *B. edulis* from cleaner regions such as Warmia and Mazury. These values exceed the median from the present study, indicating an inherent capacity of this species for Cd accumulation, independent of the level of industrialization. While the global average for edible mushrooms is estimated at 1.3925 mg·kg^−1^ d.w. [45], concentrations in *B. edulis* from China’s Yunnan province reached extreme levels ranging from 5.700 to 88.450 mg·kg^−1^ d.w. [46,47].

The analysis of lead in the present study revealed a median of 0.050 mg·kg^−1^ d.w. (max. 2.237 mg·kg^−1^ d.w.), with the highest levels found in *B. edulis* (median: 0.495 mg·kg^−1^ d.w.). This value is lower than the market averages reported by Orywal et al. [44], where mean concentrations for *Boletus edulis* and *Imleria badia* were 1.16 mg·kg^−1^ d.w. and 0.93 mg·kg^−1^ d.w., respectively. However, these levels remain far below the extreme concentrations recorded in Chile, which reached 566.8 mg·kg^−1^ d.w. [48].

The median mercury concentration was 0.055 mg·kg^−1^ d.w., with the highest levels observed in *B. edulis* (median: 0.243 mg·kg^−1^ d.w.; max: 4.240 mg·kg^−1^ d.w.), which corresponds to the reported market average of 3.04 mg·kg^−1^ d.w. [44]. The organic status had a significant impact on the purity of the raw material—products with BIO certification exhibited significantly lower Hg concentrations than those without the label (*p* = 0.007). For comparison, in the vicinity of Slovakian mines, the average Hg content in *Amanita rubescens* reached levels of 9.54–11.3 mg·kg^−1^ d.w. [43]. Additionally, Chen et al. [49] highlight the necessity of analyzing methylmercury (MeHg), which is considerably more toxic than total mercury, representing a crucial direction for future research on the safety of forest products.

The results concerning arsenic are of particular significance for safety assessment (median: 0.983 mg·kg^−1^ d.w.), with an extreme maximum of 369.048 mg·kg^−1^ d.w. recorded in *Imleria badia*. The highest median was found in the genus *Suillus* (5.364 mg·kg^−1^ d.w.). This maximum value drastically deviates from literature data; for instance, Chen et al. [49], investigating dried mushrooms from the Chinese market, reported a maximum of 132.8 mg·kg^−1^ d.w. As in *A. blazei*, while Liu et al. [50] reported a range of 0.008–57.34 mg·kg^−1^ d.w. for various species from Jilin Province. Similar conclusions are provided by studies on dried mushrooms from Turkey [51], which suggest the occurrence of incidental hyperaccumulation in specific geochemical niches, as observed in the case of *Thelephora penicillata* [52].

Significantly lower concentrations of Hg and As in mushrooms with organic certification (BIO) compared to conventional ones (*p*^UM^ of 0.007 and 0.0002, respectively) indicate the effectiveness of certification in risk mitigation. This is further supported by the findings of Gałgowska and Pietrzak-Fiećko [42], which suggest that the careful selection of harvesting areas with low anthropopressure is a key determinant of raw material purity. The pronounced differences between suppliers (e.g., Producer 7 with a HImax of 63.76 vs. Producer 4 with an HI of 0.11) demonstrate that the commercial quality of dried mushroom batches is heavily dependent on the location of forest sites. This underscores the critical need for rigorous monitoring of the raw material’s origin.

When interpreting the health risk parameters (HQ and HI), it is crucial to consider the localization of toxic elements within the mushroom structure. As discussed in the introduction, elements such as Cd, Pb, and Hg are not merely surface contaminants but are chemically sequestered within the fungal biomass, bound to structural polysaccharides or intracellular proteins [53,54,55]. While some studies on fresh mushrooms suggest that domestic processing can reduce metal loads, such measures are largely ineffective for dried products where the metals are already integrated into the dehydrated tissue [56]. Although culinary treatments such as soaking and boiling may result in minor leaching of certain water-soluble fractions, the majority of the elemental mass remains sequestered within the tissue [56,57]. Consequently, our risk assessment—based on the total concentration in the dry matter—provides a realistic yet conservative estimation. This approach accounts for the bioaccessible fraction that remains bound to the chitinous structures even after cooking, thereby ensuring that the potential health implications for the Polish consumer are not underestimated. At a moderate consumption level (3.6 g/day), the mean HI values for most species, such as chanterelles and orange birch boletes, did not exceed unity. This is consistent with the global meta-analysis by Dowlati et al. [45], which indicates that under moderate consumption, mushrooms are a safe dietary component (TTHQ for Poland = 0.978). Similar results (HI < 1) were obtained by Širić et al. [58] in studies of saprotrophic mushrooms in Croatia. Nevertheless, scenarios based on maximum values for *I. badia* (HI = 63.57) and *B. edulis* (HI = 3.36) reveal a real health hazard. As pointed out by Bucurică et al. [8] and Qin et al. [40], As and Cd are the primary determinants of risk in edible mushrooms. It should be emphasized that the risk is particularly high for children, who receive a dose of metals per kg of body weight approximately 1.5 times higher than adults [8,13]. The immense variability between producers (HI range: 0.11–63.76) proves that the commercial quality of dried mushrooms is heterogeneous and requires the implementation of systematic monitoring, with a particular focus on species from the order *Boletales*.

Chronic dietary exposure to the studied elements must be evaluated through cumulative health outcomes rather than mere regulatory compliance. Regarding Cd, accumulation in the renal cortex occurs even at low daily intakes (16–30 µg), previously deemed safe. Such levels are significantly associated with chronic kidney disease, reduced eGFR, and irreversible tubular damage [59,60,61,62]. Notably, in postmenopausal women, urinary Cd as low as ~0.3 µg/g creatinine predicts subclinical injury, suggesting that NOAEL thresholds in industrialized populations are frequently overlapped [63,64,65,66]. Similarly, Pb and Hg pose risks of cumulative neurotoxicity. For Pb, blood levels < 5–10 µg/dL correlate with permanent IQ loss and executive dysfunction, confirming no clear safe threshold [67,68]. Mechanistically, Pb disrupts glutamatergic signaling by mimicking Ca^2+^ and Zn^2+^, while methylmercury impairs neurotransmitter release and mitochondrial function. These effects span the lifespan: from neurodevelopmental delays in children to accelerated brain aging and neurodegeneration in the elderly [69,70,71,72]. Inorganic arsenic (iAs), a Group 1 carcinogen, shows causal links to skin, bladder, and lung cancers. EFSA establishes a BMDL_0.5_ as low as 0.06 µg iAs/kg bw/day, while global data attribute tens of thousands of annual cancer cases to dietary iAs [73,74,75,76,77]. With US estimates of 1500–10,000 cases per cancer type and Chinese data linking 0.55 µg/kg bw/day to significant DALYs, the elevated As in wild-harvested batches represents a tangible oncogenic risk [78,79]. Consequently, the identified ‘market-basket’ variability is a critical public health concern regarding chronic non-communicable diseases (NCDs).

### 3.1. Practical Implications

The findings of this study carry significant implications for food safety, public health policy, and environmental monitoring strategies. The distinct variation in contamination levels depending on the supplier highlights an urgent need for the implementation of rigorous control procedures by entities introducing wild-growing mushrooms into the commercial market. Quality assurance systems should encompass not only precise species identification but, more importantly, the verification of harvesting areas regarding their geochemical purity. As demonstrated in this study, organic certification—associated with a lower heavy metal burden—serves as a reliable indicator of enhanced health safety for consumers.

The significant variability in heavy metal concentrations observed across different producers and species underlines a critical gap in current food safety surveillance. In many countries, including Poland, routine monitoring of wild-harvested mushrooms is often fragmented and lacks the continuity needed to capture seasonal and geographical fluctuations. Our results demonstrate that internal quality control by producers may not be sufficient to ensure uniform consumer protection. Therefore, we propose that national food safety authorities implement a systematic, annual monitoring program for commercially available dried mushrooms. Such a targeted screening framework would allow for the identification of high-risk batches and provide the necessary data for long-term exposure assessments, ultimately bridging the gap between one-time scientific evaluations and sustainable public health policy.

The identified capacity for intensive bioaccumulation of toxic elements by selected forest species suggests the necessity of developing targeted nutritional guidelines, with particular consideration for vulnerable groups such as children and the elderly. In their diets, moderate consumption of species with known high accumulative activity should be encouraged in favor of cultivated species with more stable elemental profiles. Furthermore, the strong correlation between raw material origin and pollution levels confirms the validity of using fungal fruiting bodies as effective bioindicators in environmental monitoring. Detecting batches with extreme metal concentrations should serve as a signal for authorities to identify point-source emissions within the harvesting regions.

In parallel, it is essential to raise public awareness regarding independent mushroom foraging, emphasizing the avoidance of areas under high anthropopressure, such as the vicinity of highways or industrial facilities. These conclusions also suggest the appropriateness of introducing additional labeling requirements regarding the region of origin. This would foster a transparent market where the health quality of the product is strictly linked to environmental purity. Practical application of these insights can contribute to a tangible reduction in population exposure to toxic trace elements while promoting the safe utilization of forest resources.

### 3.2. Strengths and Limitations

The present study is characterized by several significant assets that enhance its substantive and applied value within the fields of food science and public safety. A key strength of this work is the use of a representative sample comprising 164 specimens across six mushroom species, allowing for a reliable assessment of commercial dried products available in wide circulation. Focusing the analysis on four key elements with high toxic potential—cadmium, lead, mercury, and arsenic—makes the results directly applicable to dietary risk assessment processes (ADD, HQ, HI). Of particular research value is the disclosure of drastic differences in contamination levels depending on the raw material supplier and the identification of samples with extremely high arsenic content, exceeding values previously reported in international literature. This serves as a critical signal regarding the existence of localized, heavily contaminated harvesting areas that enter the national distribution network. Furthermore, distinguishing between conventional and organic products demonstrated that certification can be a tangible factor in limiting consumer exposure to heavy metals.

At the same time, certain methodological limitations must be considered when interpreting the formulated conclusions. The analysis was based on determining the total content of elements without differentiating between their chemical forms; in the case of arsenic and mercury, this is crucial, as their toxicity to the human body is strictly dependent on speciation. Another limitation, arising from the market-based nature of sample acquisition, was the lack of direct data regarding the chemical composition of the forest substrate at the harvesting sites. This precluded the calculation of bioaccumulation factors and the definitive separation of natural species-specific predispositions for metal accumulation from the influence of point-source environmental pollution at specific producers. Furthermore, the results for lead and arsenic were found to be close to or below the limit of quantification (LOQ), which introduces a degree of uncertainty when attempting to model concentration distributions across the product population. As delineated in the Materials and Methods section, values below the LOQ were substituted with 1/2 LOQ to preserve the complete dataset and to circumvent the treatment of non-quantified concentrations as zero. However, it is important to note that this substitution may have implications for descriptive statistics, as well as for exposure and risk estimates, particularly with regard to Pb and As. Consequently, the findings concerning these elements must be interpreted with caution. Finally, the selective choice of species, while commercially representative, does not cover the full spectrum of wild-growing saprotrophic mushrooms, which narrows the generalization of the final conclusions to the most popular forest species.

## 4. Materials and Methods

### 4.1. Collection of Samples

The research material consisted of 164 samples of dried edible wild-growing mushrooms, sourced from Polish producers and acquired from retail outlets on the domestic market between January and June 2025. The vast majority of the specimens (90%, *n* = 147) originated from the 2024 harvest season, while the remaining 10% (*n* = 17) were obtained from the 2023 harvest.

The mushroom samples were procured from eight prominent Polish producers. The weight of the packages containing mushrooms ranged from 20 to 50 g.

The country of origin of the collected mushroom samples was ascertained based on the information provided on their packaging. With respect to the declared country of origin, 66% (*n* = 108) of the samples were mushrooms collected in Poland, 24% (*n* = 40) came from the European Union (EU) and outside the EU (without a specified country of origin), while 10% (*n* = 16) of the samples lacked clear information on their origin. Furthermore, 30% (*n* = 49) of the samples analysed were derived from certified ecological cultivation.

The analysis encompassed six species of edible mushrooms commonly found in the diet and trade: chanterelle (*Cantharellus cibarius*), bay bolete (*Imleria badia*), porcini (*Boletus edulis*), orange birch bolete (*Leccinum aurantiacum*), slippery jack (*Suillus luteus*), and button mushroom (*Agaricus bisporus*). The detailed characteristics of the collected research material are presented in Table S3 (Supplementary Materials).

### 4.2. Samples Preparation and Chemical Analyses

All samples were transported to the Analytical Laboratory of the Department of Environmental Health at the Medical University of Silesia in Katowice in their original unit packaging (for commercial products) to prevent secondary contamination with metalloids from polymer materials. During transport, constant relative humidity and ambient temperature were maintained, and exposure to direct sunlight was avoided.

Upon delivery to the laboratory, each sample was assigned a unique identification code containing information regarding the species, date of purchase, producer, and the presence of organic certification (BIO). Until the commencement of analytical procedures—specifically the analysis of cadmium, lead, total mercury, and arsenic content—the material was stored in glass desiccators to maintain constant mass and ensure protection against atmospheric moisture.

In the laboratory, the dried fruiting bodies were homogenized (ground to a fine powder). Each analytical portion was weighed to an accuracy of 0.5 g (with a permissible deviation of ±2%) using a PS 750/X precision balance (RADWAG, Radom, Poland). The weighed material were then transferred to Teflon vessels for subsequent acid digestion.

For the digestion process, 9 mL of ultra-pure nitric acid (HNO3, 65%; Merck, Darmstadt, Germany) and 1 mL of hydrogen peroxide (H_2_O_2_, 30%; Stanlab, Lublin, Poland) were added to each Teflon vessel containing the samples. The vessels were hermetically sealed and placed in a multi-station microwave mineralizer (ETHOS UP, Milestone, Sorisole, Italy). The mineralization was conducted according to a programmed multi-stage protocol:

Stage I (Heating): Temperature increased to 210 °C over 20 min (power: 1800 W).

Stage II (Holding): Temperature maintained at 210 °C for 15 min (power: 1800 W).

Stage III (Cooling): Ventilation and cooling phase for 30 min.

Upon completion of the digestion process, the digests were quantitatively transferred to 50 mL volumetric flasks and diluted to the mark with deionized ultrapure water.

The quantification of cadmium and lead was performed via electrothermal atomization atomic absorption spectrometry (ET-AAS) using a Savanta Sigma spectrometer (GBC Scientific Equipment, Melbourne, Australia). The system was equipped with a GF3000 graphite furnace and a PAL3000 autosampler (Analytik Jena, Jena, Germany) for automated sample delivery and optimized thermal programming.

Total mercury (THg) was determined as a representative indicator of mercury contamination. This methodology is supported by the fact that Hg in terrestrial macrofungi is predominantly sequestered in inorganic forms (Hg^2+^); meanwhile, the methylmercury (MeHg) fraction—noted for its superior bioavailability and toxicity—typically accounts for <5% of THg, often falling below the method’s limit of detection (LOD) [80,81]. THg analysis was executed using cold vapor atomic fluorescence spectrometry (CV-AFS) on a Millennium Merlin 10.025 system (PS Analytical, Orpington, UK).

Arsenic concentrations were determined using inductively coupled plasma optical emission spectrometry (ICP-OES). Measurements were conducted on an Ultima Expert LT spectrometer (HORIBA Scientific, Longjumeau, France), calibrated specifically for trace element detection in fungal matrices.

The limit of quantification (LOQ) of the method was 0.01 mg·kg^−1^ for Cd, 0.10 mg·kg^−1^ for Pb, 0.66 mg·kg^−1^ for As, and 0.002 mg·kg^−1^ for Hg, while the limit of detection (LOD) was 0.005 mg·kg^−1^ for Cd, 0.05 mg·kg^−1^ for Pb, 0.27 mg·kg^−1^ for As, and 0.001 mg·kg^−1^ for Hg. The employment of diverse analytical techniques was necessitated by the varying detection limits of the available instrumentation. A comprehensive description of the analytical methods and the specific operating conditions for each spectrometer is provided in Table 7 and Table S4 (Supplementary Materials).

The spectrometers were calibrated using certified reference solutions from AccuStandard (New Haven, CT, USA) (for Cd, serial no. 220065069; Hg, serial no. 221065171; and Pb, serial no. 220055040) and CPAchem (Stara Zagora, Bulgaria) (for As, serial no. 923794). The accuracy and precision of the analytical results were verified through spike recovery analysis using standards of known concentrations. The recovery rates and comprehensive quality control parameters are summarized in Table S5 (Supplementary Materials).

### 4.3. Health Risk Assessment

Non-cancer risks associated with metals and metaloids exposure (oral exposure) were estimated using the Average Daily Dose (ADD) formula recommended by the US Environmental Protection Agency (US EPA) [82]:

(1) $$ADD\;=\;\frac{C*\;IngrR*\;EF*\;ED}{BW*\;AT}\;[\mathrm{mg}/\mathrm{kg}/\mathrm{day}]$$

where:

ADD—Average Daily Potential Dose of As/Cd/Pb/Hg ingested via the dietary route [mg/kg/day]
C—concentration of As/Cd/Pb/Hg in the food product [mg/g]
IngR—Ingestion Rate [g/day]
EF—Exposure Frequency [365 days]
ED—Exposure Duration [70 years]
BW—Body Weight [70 kg]
AT—Averaging Time [25,550 days]

To estimate dietary exposure to the analyzed elements, an average weekly consumption of 25 g dry weight (d.w.) per capita was assumed, which corresponds to 250 g of fresh mushroom mass. In this exposure scenario, the calculated average daily consumption of edible wild forest mushrooms is 3.6 g d.w. per capita [42].

The health risk assessment for adults was performed for each element by estimating the hazard quotient (HQ) using the following formula [83]:

*HQ* = *ADD*/*RfD* (2)

Due to the lack of elemental speciation data, a conservative „worst-case scenario” was adopted. For arsenic, 100% of the total concentration was assumed to be inorganic (iAs), while for mercury, the calculations were based on the inorganic form, which predominates in terrestrial fungal species. This precautionary approach ensures that the potential health risks are not underestimated. The US EPA has established reference doses (RfD) for oral exposure, specifically: 0.0003 mg/kg/day for As [84], 0.001 mg/kg/day for Cd [85], 0.0036 mg/kg/day for Pb [86], and 0.0003 mg/kg/day for Hg [87]. To estimate the total potential non-cancer health effects due to exposure to a mixture of elements from the consumption of dried wild-growing mushrooms, a cumulative hazard index (HI) was calculated in accordance with the EPA Health Risk Assessment Guidelines [88]. The cumulative risk from concurrent exposure to multiple non-carcinogenic substances is determined by summing the individual HQ values to obtain the HI, which is interpreted in a manner analogous to the HQ index. An HQ or HI value ≤ 1 indicates negligible risk, while values > 1 suggest potential health risks [83,88]. For each sample, the relative contribution of individual metal-specific HQ values to the cumulative HI was calculated and expressed as a percentage, allowing identification of the elements contributing most to the overall non-carcinogenic risk estimate.

### 4.4. Statistical Analysis

Statistical analyses were conducted using Statistica 13.3. Quantitative data were presented as medians (Me) with the corresponding interquartile range (Q1–Q3). Qualitative data were expressed as frequencies (*n*) and percentages (%).

For statistical processing, concentrations below the limit of quantification (LOQ) were handled using the substitution method, where they were replaced by half of the LOQ value (1/2 LOQ). Specifically, the substituted values were 0.001 mg·kg^−1^, 0.005 mg·kg^−1^, 0.05 mg·kg^−1^, and 0.33 mg·kg^−1^ for the respective elements.

The normality of data distribution was verified using the Shapiro–Wilk W test, supplemented by visual inspection of histograms and distribution parameters. Given that the data did not meet the assumptions for parametric testing, non-parametric methods were used. Differences in heavy metal concentrations (Hg, Cd, Pb, As) between organic and conventional samples were assessed using the Mann–Whitney U test (*p*^U^). The relationships between metal concentrations and categorical variables, such as producer and mushroom species, were analyzed using the Kruskal–Wallis test (*p*^KW^) followed by a post hoc analysis. The strength and direction of associations between Cd, Hg, Pb, and As concentrations were evaluated using Spearman’s rank correlation coefficient (R). For all statistical analyses, the significance level was set at *α* = 0.05.

Multivariate analysis was not performed due to the small sample sizes within subgroups and the partial collinearity of factors resulting from the lack of representation for certain combinations of levels (e.g., producer × cultivation system, species × cultivation system). These constraints would have significantly limited the identifiability of model parameters.

## 5. Conclusions

The present study demonstrates that the concentrations of heavy metals in edible mushrooms are significantly influenced by species-specific traits, the certification status of the harvesting area, and the specific source of the raw material. While the analyzed mushrooms generally do not exceed safety thresholds under average consumption patterns, the distinct variability observed between species and individual producers remains a critical factor for food safety. The findings highlight that wild-growing species, particularly *Suillus luteus*, *Leccinum aurantiacum*, and *Cantharellus cibarius*, tend to accumulate higher levels of mercury and arsenic compared to cultivated control species like *Agaricus bisporus*. Furthermore, the health risk assessment, expressed through the Hazard Index (HI), confirms that although mean values remain within acceptable limits (HI < 1), incidental batches with extreme concentrations of arsenic and cadmium pose a substantial toxicological threat. This variability underscores the necessity for more rigorous monitoring of the raw material origin and the implementation of standardized quality control protocols across different producers to ensure consumer safety, especially regarding mushrooms harvested from non-certified wild areas.

## Figures and Tables

**Table 1:** Characteristic of the dried mushroom samples included in the study.

Variable	*n* (%)
Edible mushroom species	
*Cantharellus cibarius*	33 (20.12)
*Imleria badia*	47 (28.66)
*Boletus edulis*	46 (28.04)
*Leccinum aurantiacum*	15 (9.15)
*Suillus luteus*	15 (9.15)
*Agaricus bisporus*	8 (4.88)
Type of product	
certified ecological cultivation (CE)	49 (29.88)
traditional wild-growing mushrooms (T)	115 (70.12)
Producer	
Producer 1	22 (13.41)
Producer 2	8 (4.88)
Producer 3	8 (4.88)
Producer 4	23 (14.02)
Producer 5	8 (4.88)
Producer 6	16 (9.76)
Producer 7	48 (29.27)
Producer 8	31 (18.90)

**Table 2:** Mercury (Hg) concentrations in edible mushrooms according to selected material characteristics.

Variable	Me (Q1–Q3)	Minimum	Maximum	Statistical Analysis
Type of product				
CE	0.029 (0.021–0.074)	0.001 *	0.256	CE ≠ T
T	0.070 (0.019–0.218)	0.001 *	4.240	(*p*^UM^ = 0.007)
Edible mushroom species				
*Cantharellus cibarius*	0.018 (0.001 *–0.024)	0.001 *	0.202	C ^1^
*Imleria badia*	0.034 (0.021–0.104)	0.001 *	0.767	B ^1^
*Boletus edulis*	0.243 (0.099–0.859)	0.046	4.240	A ^1^
*Leccinum aurantiacum*	0.065 (0.051–0.086)	0.032	0.134	AB ^1^
*Suillus luteus*	0.064 (0.034–0.078)	0.018	0.119	B ^1^
*Agaricus bisporus*	0.001 (0.001 *–0.003)	0.001 *	0.004	C ^1^
Producer				
Producer 1	0.026 (0.021–0.041)	0.017	0.661	AB ^1^
Producer 2	0.155 (0.042–0.515)	0.027	1.177	AC ^1^
Producer 3	0.053 (0.021–0.105)	0.018	0.134	AC ^1^
Producer 4	0.072 (0.054–0.106)	0.028	0.341	AC ^1^
Producer 5	0.157 (0.088–0.271)	0.060	0.480	BC ^1^
Producer 6	0.825 (0.064–1.946)	0.016	4.240	C ^1^
Producer 7	0.019 (0.001 *–0.088)	0.001 *	1.958	A ^1^
Producer 8	0.043 (0.021–0.091)	0.001 *	0.256	AC ^1^

Note. Me—median; Q1—first quartile; Q3—third quartile; CE—certified organic cultivation; T—traditional wild-growing mushrooms; *—concentrations below the limit of quantification (LOQ); *p*^UM^—Mann–Whitney U test; ^1^—Values followed by the same letter within a row/column do not differ significantly (*p* > 0.05), while different letters indicate statistically significant differences (*p* ≤ 0.05) based on the Kruskal–Wallis test with post hoc analysis. Multi-letter designations (e.g., “AB”, “AC”) indicate that the group does not differ significantly from any of the groups marked with those letters.

**Table 3:** Cadmium (Cd) concentrations in edible mushrooms according to selected material characteristics.

Variable	Me (Q1–Q3)	Minimum	Maximum	Statistical Analysis
Type of product				
CE	0.95 (0.09–1.58)	0.040	2.90	CE = T
T	0.52 (0.17–1.24)	0.005 *	9.86	(*p*^UM^ = 0.25)
Edible mushroom species				
*Cantharellus cibarius*	0.17 (0.08–0.19)	0.04	0.22	A ^1^
*Imleria badia*	0.95 (0.57–1.56)	0.23	3.01	B ^1^
*Boletus edulis*	1.30 (0.73–1.88)	0.14	9.86	B ^1^
*Leccinum aurantiacum*	0.68 (0.55–1.11)	0.38	1.56	B ^1^
*Suillus luteus*	0.09 (0.07–0.13)	0.005 *	1.48	A ^1^
*Agaricus bisporus*	0.040 (0.025–0.050)	0.005 *	0.07	A ^1^
Producer				
Producer 1	0.09 (0.07–1.93)	0.04	5.71	AB ^1^
Producer 2	0.96 (0.51–1.46)	0.32	2.71	AC ^1^
Producer 3	1.19 (0.42–8.06)	0.25	9.86	AC ^1^
Producer 4	0.79 (0.60–1.49)	0.38	3.01	C ^1^
Producer 5	1.475 (0.895–2.080)	0.39	2.61	C ^1^
Producer 6	0.555 (0.380–0.945)	0.23	2.63	AC ^1^
Producer 7	0.16 (0.075–0.20)	0.005 *	2.49	B ^1^
Producer 8	1.33 (0.95–1.69)	0.47	2.90	C ^1^

Note. Me—median; Q1—first quartile; Q3—third quartile; CE—certified organic cultivation; T—traditional wild-growing mushrooms; *—concentrations below the limit of quantification (LOQ); *p*^UM^—Mann–Whitney U test; ^1^—Values followed by the same letter within a row/column do not differ significantly (*p* > 0.05), while different letters indicate statistically significant differences (*p* ≤ 0.05) based on the Kruskal–Wallis test with post hoc analysis. Multi-letter designations (e.g., “AB”, “AC”) indicate that the group does not differ significantly from any of the groups marked with those letters.

**Table 4:** Lead (Pb) concentrations in edible mushrooms according to selected material characteristics.

Variable	Me (Q1–Q3)	Minimum	Maximum	Statistical Analysis
Type of product				
CE	0.05 * (0.05–1.026)	0.05 *	2237	CE = T
T	0.05 * (0.05–0.233)	0.05 *	1878	(*p*^UM^ = 0.18)
Edible mushroom species				
*Cantharellus cibarius*	0.05 * (0.05–0.05)	0.05 *	1.878	A ^1^
*Imleria badia*	0.05 * (0.05–0.209)	0.05 *	1.094	A ^1^
*Boletus edulis*	0.495 (0.163–1.306)	0.05 *	2.237	B ^1^
*Leccinum aurantiacum*	0.183 (0.05 *–0.286)	0.05 *	0.636	AB ^1^
*Suillus luteus*	0.05 * (0.05–0.05)	0.05 *	0.317	A ^1^
*Agaricus bisporus*	0.05 * (0.05–0.05)	0.05 *	0.05 *	A ^1^
Producer				
Producer 1	0.05 * (0.05–0.05)	0.05 *	0.566	AB ^1^
Producer 2	0.128 (0.05–0.433)	0.05 *	0.700	AC ^1^
Producer 3	0.363 (0.05–0.873)	0.05 *	1.770	AC ^1^
Producer 4	0.163 (0.05–0.289)	0.05 *	0.877	BC ^1^
Producer 5	0.185 (0.122–0.269)	0.05 *	0.569	AC ^1^
Producer 6	0.128 (0.05–0.264)	0.05 *	1.376	AC ^1^
Producer 7	0.05 (0.05–0.05)	0.05 *	1.878	A ^1^
Producer 8	0.371 (0.05–1.852)	0.05 *	2.237	C ^1^

Note. Me—median; Q1—first quartile; Q3—third quartile; CE—certified organic cultivation; T—traditional wild-growing mushrooms; *—concentrations below the limit of quantification (LOQ); *p*^UM^—Mann–Whitney U test; ^1^—Values followed by the same letter within a row/column do not differ significantly (*p* > 0.05), while different letters indicate statistically significant differences (*p* ≤ 0.05) based on the Kruskal–Wallis test with post hoc analysis. Multi-letter designations (e.g., “AB”, “AC”) indicate that the group does not differ significantly from any of the groups marked with those letters.

**Table 5:** Arsenic (As) concentrations in edible mushrooms according to selected material characteristics.

Variable	Me (Q1–Q3)	Minimum	Maximum	Statistical Analysis
Type of product				
CE	0.33 * (0.33–1.051)	0.33 *	2.811	CE ≠ T
T	1.232 (0.33–4.112)	0.33 *	369.048	(*p*^UM^ = 0.0002)
Edible mushroom species				
*Cantharellus cibarius*	1.217 (1.005–1.488)	0.33 *	2.811	A ^1^
*Imleria badia*	0.33 * (0.33–1.427)	0.33 *	369.048	B ^1^
*Boletus edulis*	0.33 * (0.33–3.803)	0.33 *	12.223	AB ^1^
*Leccinum aurantiacum*	1.321 (0.33–2.078)	0.33 *	2.850	AB ^1^
*Suillus luteus*	5.364 (4.412–8.260)	2.067 *	11.014	C ^1^
*Agaricus bisporus*	0.33 * (0.33–0.33)	0.33 *	0.33 *	B ^1^
Producer				
Producer 1	1.337 (1.030–2.068)	0.33 *	2.811	AB ^1^
Producer 2	0.33 * (0.33–6.329)	0.33 *	12.223	ABC ^1^
Producer 3	0.33 * (0.33–1.262)	0.33 *	6.824	ABC ^1^
Producer 4	0.33 * (0.33–1.663)	0.33 *	2.850	ABC ^1^
Producer 5	2.822 (0.33–4.504)	0.33 *	5.796	ABC ^1^
Producer 6	1.807 (0.33–6.856)	0.33 *	8.567	AB ^1^
Producer 7	1.402 (0.874–5.674)	0.33 *	369.048	B ^1^
Producer 8	0.33 * (0.33–0.33)	0.33 *	2.779	C ^1^

Note. Me—median; Q1—first quartile; Q3—third quartile; CE—certified organic cultivation; T—traditional wild-growing mushrooms; *—concentrations below the limit of quantification (LOQ); *p*^UM^—Mann–Whitney U test; ^1^—Values followed by the same letter within a row/column do not differ significantly (*p* > 0.05), while different letters indicate statistically significant differences (*p* ≤ 0.05) based on the Kruskal–Wallis test with post hoc analysis. Multi-letter designations (e.g., “AB”, “ABC”) indicate that the group does not differ significantly from any of the groups marked with those letters.

**Table 6:** Spearman’s rank correlation coefficients between the concentrations of the analyzed metals.

Variable	Spearman’s Rank Correlation Coefficients (R)			
	Hg [mg·kg^−1^]	Cd [mg·kg^−1^]	Pb [mg·kg^−1^]	As [mg·kg^−1^]
Hg [mg·kg^−1^]	1.000	0.508	0.466	0.215
Cd [mg·kg^−1^]	0.508	1.000	0.533	−0.261
Pb [mg·kg^−1^]	0.466	0.533	1.000	−0.082
As [mg·kg^−1^]	0.215	−0.262	−0.082	1.000

**Table 7:** Method description and measuring conditions of the spectrometers ICP-OES, ET-AAS, and CV-AFS.

Element	As	Cd	Pb	Hg
Analytical methods	ICP-OES	ET-AAS		CV-AFS
Line [nm]	197.197	228.8	217.0	254
Calibration range [mg/L]; [µg/L] *	0.1–5.0	5.00–20.00	10.00–40.00	0.00–20.00
Calibration type	maximum	least squares		least squares
Calibration of failure criteria: R^2^	≥0.995			
RSD for standards [%]	<5%	<10%		<10%
Slit width [nm]	-	0.50	1.00	-
Lamp current [mA]	-	3.00	5.00	-
Background correction	-	deuterium		-
Sample volume [µL]	-	20.00	16.00	-
Gas flow (Ar 99,999%) [L/min]	12–13	3		2.50
Radio frequency power [W]	1000	-		-
Type of nebulizer	Teflon	-		-
Peristaltic pump speed [rpm] **	15	-		100 [%]

* [mg/L] for ICP-OES; [µg/L] for ET-AAS and CV-AFS. ** % for CV-AFS.

## Data Availability

The datasets generated during the current study are available from the corresponding author on reasonable request.

## Supplementary Materials

The following supporting information can be downloaded at: https://www.mdpi.com/article/10.3390/molecules31111865/s1. Table S1. Average daily dose and health risks associated with multi-trace element exposure in adults from consumption of dried wild edible mushroom fruiting bodies (intake rate—3.6 g/day); Table S2. Average daily dose and health risks associated with multi-trace element exposure in adults from consumption of dried wild edible mushroom fruiting bodies varying by producer; Table S3. Characteristics of mushroom samples; Table S4. Cadmium and lead cuvette program; Table S5. Characteristics of the method.

## Abbreviations

The following abbreviations are used in this manuscript:

AB	*Agaricus bisporus*
ADD	Average Daily Dose
As	Arsenic
BE	*Boletus edulis*
CC	*Cantharellus cibarius*
Cd	Cadmium
CE	certified ecological cultivation
d.w.	dry weight
Hg	Mercury
HI	Hazard Index
HQ	Hazard Quotient
iAs	Inorganic arsenic
IB	*Imleria badia*
LA	*Leccinum aurantiacum*
LOD	Limit of Detection
LOQ	Limit of Quantification
MeHg	Methylmercury
NCDs	Non-communicable diseases
Pb	Lead
RfD	Reference Dose
SL	*Suillus luteus*
T	traditional wild-growing mushrooms
