# Supplementary material for: Evaluation of Toxic Elements in Commercial Dried Mushrooms from the Polish Market: Exposure Assessment and Health Risk Characterization
Source: Molecules. 2026 May 29;31(11):1865. doi: 10.3390/molecules31111865 (PMC13258499; doi:10.3390/molecules31111865)
Supplement: Supplementary file 1 [file molecules-31-01865-s001.zip › molecules-4309261-supplementary.pdf]

Table S1 Average daily dose and health risks associated with multi-trace element exposure in adults from consumption of dried wild edible mushroom fruiting bodies (intake rate – 3.6 g/day)

Variable	Parameters of concentration	As			Cd			Hg			Pb			HI
		C [mg·kg^{-1}]	ADD	HQ	C [mg·kg^{-1}]	ADD	HQ	C [mg·kg^{-1}]	ADD	HQ	C [mg·kg^{-1}]	ADD	HQ	
Edible mushroom species														
Cantharellus cibarius	Mean	1.217	0.00006	0.2086	0.170	0.00001	0.0087	0.018	0.0000009	0.0031	0.050	0.0000026	0.0007	0.2212
	Max	2.811	0.00014	**0.4819**	0.220	0.00001	0.0113	0.202	0.00001	0.0346	1.878	0.00010	0.0268	0.5547
Imleria badia	Mean	0.330	0.00002	0.0566	0.950	0.00005	0.0489	0.034	0.0000017	0.0058	0.050	0.0000026	0.0007	0.1120
	Max	369.048	0.01898	**63.2654**	3.01	0.00015	0.1548	0.77	0.00004	0.1315	1.09	0.00006	0.0156	**63.5673**
Boletus edulis	Mean	0.330	0.00002	0.0566	1.300	0.00007	0.0669	0.243	0.000012	0.0417	0.495	0.00003	0.0071	0.1722
	Max	12.223	0.00063	**2.0954**	9.86	0.00051	0.5071	4.24	0.00022	0.7269	2.24	0.00012	0.0320	**3.3613**
Leccinum aurantiacum	Mean	1.321	0.00007	0.2265	0.680	0.00003	0.0350	0.065	0.000003	0.0111	0.183	0.00001	0.0026	0.2752
	Max	2.850	0.00015	0.4886	1.560	0.00008	0.0802	0.134	0.00001	0.0230	0.636	0.00003	0.0091	0.6009
Suillus luteus	Mean	5.364	0.00028	0.9195	0.090	0.000005	0.0046	0.064	0.000003	0.0110	0.050	0.000003	0.0007	0.9359
	Max	11.014	0.00057	**1.8881**	1.480	0.00008	0.0761	0.119	0.00001	0.0204	0.317	0.00002	0.0045	**1.9892**
Agaricus bisporus	Mean	0.330	0.00002	0.0566	0.040	0.000002	0.0021	0.001	0.0000001	0.0002	0.050	0.000003	0.0007	0.0595
	Max	0.330	0.00002	0.0566	0.070	0.000004	0.0036	0.004	0.0000002	0.0007	0.050	0.000003	0.0007	0.0616
Type of product														
ecological	Mean	0.330	0.00002	0.0566	0.950	0.00005	0.0489	0.029	0.0000015	0.0050	0.050	0.0000026	0.0007	0.1111
	Max	2.811	0.00014	0.4819	2.900	0.00015	0.1491	0.256	0.00001	0.0439	2.237	0.00012	0.0320	0.7069
traditional	Mean	1.232	0.00006	0.2112	0.520	0.00003	0.0267	0.070	0.000004	0.0120	0.050	0.000003	0.0007	0.2507
	Max	369.048	0.01898	**63.2654**	9.860	0.00051	0.5071	4.240	0.000218	0.7269	1.878	0.00010	0.0268	**64.5261**

Note. Max – maximum concentration; C – concentration; ADD – Average Daily Dose; HQ - Hazard Quotient; HI - Hazard Index

Table S2 Average daily dose and health risks associated with multi-trace element exposure in adults from consumption of dried wild edible mushroom fruiting bodies varying by producer

Producer	Parameters of concentration	As			Cd			Hg			Pb			HI
		C [mg·kg⁻¹]	ADD	HQ	C [mg·kg⁻¹]	ADD	HQ	C [mg·kg⁻¹]	ADD	HQ	C [mg·kg⁻¹]	ADD	HQ	
Producer 1	Mean	1.337	0.00007	0.2292	0.090	0.000005	0.0001	0.026	0.000001	0.0045	0.050	0.000003	0.0007	0.2344
	Max	2.811	0.00014	0.4819	5.710	0.00029	0.2937	0.661	0.00003	0.1133	0.566	0.00003	0.0081	0.8969
Producer 2	Mean	0.330	0.00002	0.0566	0.960	0.00005	0.0494	0.155	0.00001	0.0266	0.128	0.00001	0.0018	0.1343
	Max	12.223	0.00063	**2.0954**	2.710	0.00014	0.1394	1.177	0.00006	0.2018	0.700	0.00004	0.0100	**2.4465**
Producer 3	Mean	0.330	0.00002	0.0566	1.190	0.00006	0.0612	0.053	0.000003	0.0091	0.363	0.00003	0.0052	0.1320
	Max	6.824	0.00035	**1.1698**	9.860	0.00051	0.5071	0.134	0.00001	0.0230	1.770	0.00009	0.0253	**1.7252**
Producer 4	Mean	0.330	0.00002	0.0566	0.790	0.00004	0.0406	0.072	0.000004	0.0123	0.163	0.00001	0.0023	0.1119
	Max	2.850	0.00015	0.4886	3.010	0.00015	0.1548	0.341	0.00002	0.0585	0.877	0.00005	0.0125	0.7144
Producer 5	Mean	2.822	0.00015	0.4838	1.475	0.00008	0.0759	0.157	0.000008	0.0269	0.185	0.00001	0.0026	0.5892
	Max	5.796	0.00030	0.9936	2.610	0.00013	0.1342	0.480	0.00002	0.0823	0.569	0.00003	0.0081	**1.2182**
Producer 6	Mean	1.807	0.00009	0.3098	0.555	0.00003	0.0285	0.825	0.000042	0.1414	0.128	0.00001	0.0018	0.4816
	Max	8.567	0.00044	**1.4686**	2.630	0.00014	0.1353	4.240	0.00022	0.7269	1.376	0.00007	0.0197	**2.3504**
Producer 7	Mean	1.402	0.00007	0.2403	0.160	0.00001	0.0082	0.019	0.000001	0.0033	0.050	0.000003	0.0007	0.2525
	Max	369.048	0.01898	**63.2654**	2.490	0.00013	0.1281	1.958	0.00010	0.3357	1.878	0.00010	0.0268	**63.7559**
Producer 8	Mean	0.330	0.00002	0.0566	1.330	0.00007	0.0684	0.043	0.000002	0.0074	0.371	0.00002	0.0053	0.1376
	Max	2.779	0.00014	0.4764	2.900	0.00015	0.1491	0.256	0.00001	0.0439	2.237	0.00012	0.0320	0.7014

Note. Max – maximum concentration; C – concentration; ADD – Average Daily Dose; HQ - Hazard Quotient; HI - Hazard Index

Table S3 Characteristics of mushroom samples

Producer	Mushroom species	n	Type of product[A]
Producer 1	*Cantharellus cibarius*	16	CE
	Imleria badia	2	CE
	Boletus edulis	4	T
Producer 2	*Imleria badia*	4	T
	Boletus edulis	4	
Producer 3	*Imleria badia*	4	T
	Boletus edulis	4	
Producer 4	*Imleria badia*	4	T
	Boletus edulis	4	
	Leccinum aurantiacum	15	
Producer 5	*Imleria badia*	4	T
	Boletus edulis	4	
Producer 6	*Imleria badia*	8	T
	Boletus edulis	8	
Producer 7	*Boletus edulis*	4	T
	Suillus luteus	15	
	Agaricus bisporus	8	
	Cantharellus cibarius	17	
	Imleria badia	4	
Producer 8	*Imleria badia*	17	CE
	Boletus edulis	14	
	Total	164	

Note. a CE - certified ecological cultivation; T – traditional wild-growing mushrooms

Table S4 Cadmium and lead cuvette program

Element	Step	Cuvette program			
		Final temperature [°C]	Rise time [s]	Maintenance time [s]	Gas type
Cd	1	50	1.0	1.0	inert
			Injection		
	3	100	10.0	15.0	inert
	4	120	5.0	10.0	inert
	5	300	10.0	7.0	inert
	6	300	1.0	1.0	inactive
	7	1800	1.0	3.0	inactive
	8	2100	1.0	2.0	inert
Pb	1	50	1.0	1.0	inert
			Injection		
	3	100	10.0	15.0	inert
	4	120	15.0	20.0	inert
	5	600	10.0	7.0	inert
	6	600	1.0	1.0	inactive
	7	2200	0.8	2.0	inactive
	8	2200	1.0	2.0	inert

Tabele S5 Characteristics of the method

Parameter	As	Cd	Pb	Hg
R^2 value	0.997	0.998	0.999	0.999
Recovery [%]	95-109	84-113	88-106	83-96
Relative Standard Deviation – RSD [%]	< 10%	< 20 %	< 20 %	< 10 %
LOD [$mg \cdot kg^{-1}$]	0.27	0.005	0.05	0.001
LOQ [$mg \cdot kg^{-1}$]	0.66	0.01	0.10	0.002
